# Host-Specific and pH-Dependent Microbiomes of Copepods in an Extensive Rearing System

**DOI:** 10.1371/journal.pone.0132516

**Published:** 2015-07-13

**Authors:** Alf Skovgaard, Josue Leonardo Castro-Mejia, Lars Hestbjerg Hansen, Dennis Sandris Nielsen

**Affiliations:** 1 Department of Veterinary Disease Biology, University of Copenhagen, DK-1870, Frederiksberg, Denmark; 2 Department of Food Science, University of Copenhagen, DK-1958, Frederiksberg, Denmark; 3 Department of Environmental Science, Aarhus University, DK-4000, Roskilde, Denmark; National Taiwan Ocean University, TAIWAN

## Abstract

Copepods are to an increasing extent cultivated as feed for mariculture fish larvae with variable production success. In the temperate climate zone, this production faces seasonal limitation due to changing abiotic factors, in particular temperature and light. Furthermore, the production of copepods may be influenced by biotic factors of the culture systems, such as competing microorganisms, harmful algae, or other eukaryotes and prokaryotes that may be non-beneficial for the copepods. In this study, the composition of bacteria associated with copepods was investigated in an extensive outdoor copepod production system. Light microscopy and scanning electron microscopy revealed that bacteria were primarily found attached to the exoskeleton of copepods although a few bacteria were also found in the gut as well as internally in skeletal muscle tissue. Through 16S rRNA gene-targeted denaturing gradient gel electrophoresis (DGGE) analysis, a clear difference was found between the microbiomes of the two copepod species, *Acartia tonsa* and *Centropages hamatus*, present in the system. This pattern was corroborated through 454/FLX-based 16S rRNA gene amplicon sequencing of copepod microbiomes, which furthermore showed that the abiotic parameters pH and oxygen concentration in rearing tank water were the key factors influencing composition of copepod microbiomes.

## Introduction

Copepods are key organisms in the marine pelagic ecosystem where they play an important role as secondary producers channelling organic carbon from planktonic primary producers to higher trophic levels [1] and copepods compose the natural diet for most marine fish larvae [2]. In addition, copepods play an important role by providing microenvironments for associated bacteria, thereby facilitating bacterial growth and activity and increasing bacterial diversity and migration [3–5].

Copepods are valuable as live feed for marine fish larvae produced in mariculture [6] and copepods today are being produced in large-scale culture systems for this purpose [7]. It is of great importance that copepods used as live feed do not transmit parasites or harmful microorganisms to the fish larvae. This circumstance makes the use of wild-caught copepods problematic because copepods function as intermediate hosts for several species of fish parasites that are unwanted in a fish larvae production system (such as trematodes and nematodes [8, 9]). These parasites are not present in cultivated copepods since the hosts needed for fulfilment of the parasite’s life cycle (typically an intermediate invertebrate host and a fish final host) are normally absent in the culture systems. A large number of protozoan parasites also thrive in marine planktonic copepods [10, 11], but even though many of these parasites can have severe effects on their copepod host, very few parasite species have been observed in cultured copepods, suggesting that this issue probably does not compose any severe problem for large-scale copepod production. Furthermore, no protozoan parasites of copepods have yet proved pathogenic to fish or fish larvae.

Hence, metazoan and protozoan pathogens are generally not considered an issue of concern in cultivated copepods. On the other hand, both wild and cultured copepods are widely colonized by bacteria [12–14]. The human-pathogenic bacteria *Vibrio cholera* is commonly found associated with coastal marine copepods [13, 15, 16]. For this reason, and because copepods are known to function as bacterial ‘hot-spots’ in the sea [4, 17], a considerable research effort has been dedicated to understanding the interaction between marine copepods and their associated bacteria (i.e. their microbiome). This research has mainly focused on specific species or classes of bacteria with human pathogenic potential [13, 16, 18], whereas less effort has been put into examining the composition of the entire microbiome of marine copepods. Therefore, it remains largely unknown whether the copepod microbiome may contain bacteria that are pathogenic to either the copepods themselves or, possibly, the fish larvae feeding upon them.

Accordingly, both from an ecology and aquaculture point of view it is important to gain more knowledge about bacteria associated with copepods. Knowledge on copepod microbiomes has so far mainly been based on scanning electron microscopy [19] and culture-dependent methods [12, 20], and more recently, PCR, cloning, and denaturing gradient gel electrophoresis (DGGE) has also been employed [21–23].

The early studies using culture-dependent techniques revealed bacteria of several classes to be commonly associated with copepods in cultures [12, 20, 24] and in the ocean [13]. Bacteria that have been identified through microbiological cultivation techniques have pertained to the classes Gammaproteobacteria (genera *Vibrio*, *Photobacterium*, *Alteromonas*, *Pseudomonas*, and *Aeromonas* [12, 13, 20]); Betaproteobacteria (*Chromobacterium* [20]); and *Cytophaga*/*Flavobacterium* and *Corynebacterium* of the phyla Bacteriodites and Actinobacteria, respectively [12].

The association of *Vibrio* spp. with wild and cultured copepods has been demonstrated repeatedly [25] and this association has also been confirmed by use of DNA-probes [16]. Recent surveys based on PCR and clone libraries have confirmed the presence of Gammaproteobacteria in association with copepods [3, 22, 23]. Moreover, bacteria of the class Alphaproteobacteria, not detected in the culture-dependent studies, have been shown to be among the numerically dominant groups in copepod microbiomes, and in particular the family Rhodobacteraceae is widely associated with copepods [3, 21–23, 26]. Molecular techniques have also confirmed the presence of Bacteroidia, Flavobacteria, and Actinobacteria in low abundance, and have added considerably to the recognized bacterial species/genotype diversity of the microbiomes of marine copepods [22, 27]. Subsequently, the classes Deltaproteobacteria [3, 22], Verrucomicrobiae (phylum Verrucomicrobia), Bacilli and Clostridia (phylum Firmicutes), and Sphingobacteria (phylum Bacteriodites) have also been found to occur in low abundance [3, 27].

The ecological role of bacteria associated with copepods is complex. Copepod carcasses constitute a significant source of nutrients for bacteria in the ocean [28], but bacteria may also utilize live copepods as substrate and/or may exploit organic matter produced by the host. Some bacteria typically associated with marine copepods, such as *Vibrio* spp. and *Sphingomonas* sp., express chitinases and are able to utilize chitin as source for carbon and nitrogen [29–31]. This suggests that some bacteria have adapted for growth in association with the copepod exoskeleton, but the possible effect on copepod physiology is unknown. Some uncertainties exist with respect to whether bacteria associated with copepods are “host-specific” in the sense that different copepod species in the same environment host quantitatively or qualitatively different bacteria communities. It has been shown that bacterial diversity associated with four North Sea copepod species is invariant with copepod species and growth season [26]. Another study, on the other hand, revealed a potential difference in bacterial diversity across two copepod species from the Arctic [3].

While a number of studies have delivered valuable knowledge on the diversity of bacteria associated with copepods, only limited knowledge exists on the complete microbiome of live, marine copepods, the development in copepod microbiomes and their possible dependence on host species and growth conditions. The current study aims at investigating microbiomes associated with copepods in a turbot larvae (*Scophthalmus maximus)* production facility. The composition of the bacterial community was compared across copepod species (*Acartia tonsa* and *Centropages hamatus*) in the cultivation system by use of denaturing gradient gel electrophoresis (DGGE) and 454/FLX-based 16S rRNA gene amplicon sequencing (454-pyrosequencing), and additional observations were made on development of bacterial community structure over time. Furthermore, the actual sites of bacterial presence in/on copepods were investigated by use of scanning electron microscopy (SEM) and in-situ hybridization.

## Experimental Procedures

### Experimental setup

The study site was the turbot fry farm Maximus A/S located in Northern Jutland, Denmark (56°49'1.68"N, 8°31'46.13"E), specialized in producing juvenile turbot of a size up to approximately 5 g. The first-feeding turbot larvae were fed copepod nauplii produced in large outdoor concrete tanks. This study was part of a larger field campaign at the fish farm in August-September 2012. The campaign had the overall goal to investigate the possibility of enhancing production in culture tanks by enriching phytoplankton with inorganic nutrients, which should in turn lead to increased copepod production and, thereby, better food supply for the turbot larvae. Detailed information on the copepod rearing system including measurements of biological and chemical data related to the culturing system is reported elsewhere [32]. In the present field campaign, copepod-rearing tanks were manipulated in three parallel treatments: Copepods were produced in six tanks with a capacity of 280 m^3^ and three tanks with a capacity of 50 m^3^. The tanks were filled with 50-μm filtered, 29‰ salinity seawater pumped from the nearby bay (Visby Bredning, Limfjorden). The tanks were divided into three treatment groups of each 2 large and one small tank. The treatments were 1) a control treatment in which the three tanks received only filtered seawater, 2) a full dose treatment in which the tanks received a single, large dose of nutrients, and 3) a pulse dose treatment in which tanks received several smaller doses of nutrients. The commercial fertilizer Kristalon PK643K from YARA International ASA (Oslo, Norway) was used. Nutrients were initially added the 8^th^ of August 2012. The full dose treatment tanks received nutrient corresponding to a final concentration of 85 μmol L^-1^. The pulse dose treatment tanks received 2 additional doses aiming at reaching a final concentration of 25 μmol L^-1^ *per* dose. Tanks were left for two days for the phytoplankton community to become established and copepods were then added to all nine tanks in order to achieve a complete plankton food web. These copepods originated from a purpose-made copepod stock culture previously established in an adjacent artificial lagoon. The tanks were left untouched for another three days to let copepods commence reproduction and sampling was started on August 13^th^ 2012. No vertebrates were sampled or otherwise manipulated during this study. Maximus A/S operated under a licence from The Ministry of Food, Agriculture and Fisheries of Denmark. No specific permissions were required for the underlying work at the fish farm, whose general work was regulated by the Animal welfare act of The Ministry of Food, Agriculture and Fisheries of Denmark

### Sampling of copepods

The zooplankton communities in the tanks were comprised almost exclusively of the two calanoid copepod species *Centropages hamatus* and *Acartia tonsa*. Copepods were collected from growth tanks by use of a hand-operated plankton net with a mesh size of 200 μm. Copepods were brought directly to an on-site laboratory, adult and late copepodite stage copepods were isolated under a dissection microscope, and the two species were separated. For bacterial DNA analysis, copepods were isolated and washed three times in freshly 0.2 μm-filtered tank water and 20–25 copepods per sample were transferred to sterile 1.5-ml Eppendorf tubes. Residual water was removed from the tubes with a drawn-out Pasteur pipette carefully avoiding sucking up any copepods. Samples prepared for DNA-analysis were placed immediately at −20°C. Other samples were preserved in Davidson’s fixative for subsequent preparation for SEM or thin sectioning. Samples of *C*. *hamatus* were taken from all tanks in the experimental setup in the beginning of the campaign and once again two weeks later. Samples of *A*. *tonsa* were only taken at the beginning of the campaign, since this species was not present in large enough numbers to achieve comparable samples from all tanks. *Acartia tonsa* eventually became the dominating species in the full dose tanks, but this shift in species composition occurred only after the sampling campaign had finished [32]. In addition, two samples were taken from an indoor, intensive *A*. *tonsa* culture system a Roskilde University, Denmark, on September 12^th^ 2012. These samples were included to investigate whether any tight species specificity would be present would occur between certain bacteria and their copepod host.

### SEM and semi-thin sectioning/in-situ hybridization

Copepods from both sampling sites were fixed in Davidson’s fixative for 24 h and subsequently stored in 70% ethanol in order to qualitatively visualize associated bacteria. Samples for SEM were dehydrated in a graded ethanol series with final dehydration in hexamethyldisilazane [33]. Copepods were then mounted individually on a stub covered with double adhesive tape and viewed in a Hitachi S-3500N scanning electron microscope (operating at 5 kV) located at the Natural History Museum of Denmark.

For semi-thin sectioning, copepods were first transferred to drops of warm Histogel (Thermo Scientific, Denmark) placed on microscope slides. When the Histogel had solidified, the pieces of the gel containing copepods were cut out, placed in tissue processing cassettes and dehydrated in a graded ethanol series (15 min at each step). Dehydration was finished with two 15-minute baths in xylene and the Histogel pellets were then embedded in paraffin and 4 μm sections were cut using a Leica RM2135 microtome (Leica Microsystems, Germany). Sections were mounted on positively charged glass slides and dried at 40°C for 24 h.

Preparation for *in-situ* hybridization followed previously described procedures [34] with minor modifications [35]. Briefly, Slides were deparaffinised in xylene and rehydrated through a graded ethanol series. The slides were then incubated in 0.1% active DEPC-PBS (phosphate buffer saline, pH = 7.4) for 10 min, washed in PBS for 5 min and rinsed in Milli-Q water for 10 s. Slides were then re-rehydrated in two steps of ethanol (3 min at each step) and air-dried at 37°C for 20–30 min. For hybridization, 150 ml of hybridization buffer containing EUB338 probe [36] or non-338 probe [35] at 5 ng/ml in wash buffer with 20% formamide was added to each slide, which were then incubated at 37°C overnight in a humidified chamber. Probes were labelled with Cy3 at the 5’ end (TAG Copenhagen A/S, Denmark). The hybridization buffer/probe mixture was the following day gently poured off and slides were incubated for 20 min in pre-warmed hybridization buffer (37°C) containing 20% formamide, but no probe. Finally, slides were rinsed briefly in pre-warmed Milli-Q water, air dried for 30 min while kept in darkness, and mounted with Vectashield mounting medium with DAPI (Vector Laboratories, Inc. Burlingame, CA, USA). Tissue sections were viewed using an Olympus VANOX-T epifluorescence microscope (Olympus, Japan) equipped with an Olympus UC30 camera and appropriate filter sets [35]. For visualization of stained bacteria on tissue sections, photographs made using filter sets for the Cy3-probe and the green autofluorescence, respectively, were super-imposed by use of Adobe Photoshop CS5 software.

### Extraction of Bacterial DNA

The bacteria communities associated with copepods were analysed both through DGGE profiling and by 454-pyrosequencing. For DNA extraction, samples were first thawed and then homogenized by use of a battery-driven Kontes Pellet Pestle (VWR—Bie & Berntsen, Denmark) using sterile disposable pestles. DNA was extracted by use of the QIAamp DNA Mini Kit (QIAGEN Denmark) following the manufacturer’s instructions. DNA extract was finally eluted into 30 μl of sterile, DNase-free water.

### DGGE

The V3 region of the bacterial 16S rRNA gene was amplified by PCR using the universal primer set PRBA338f and PRUN518r [37], manufactured by (Eurofins MWG Operon, Ebensburg, Germany). PCR reactions were set up in a benchtop hood that had been UV-irradiated for minimum 3 h. Reactions were done in 50 μl reactions in single tubes (i.e. not strips, to minimize the risk of cross-contaminating samples) with 5 μl BIOTAQ 10x NH_4_ Reaction Buffer, 1.5 μl 50 mM MgCl2, 8 μl 10 mM dNTP, 5 μl forward primer and 5 μl reverse primer at 10 mM, 5 μl template, 0.5 μl BIOTAQ DNA Polymerase (Bioline Reagents Ltd, UK), and DNAse-free water up to 50 μl. PCR was run on a Biometra T3 Thermocycler with initial denaturation at 95°C for 5 min, followed by 30 cycles of denaturation at 95°C for 30 s, annealing at 60°C for 30 s, and extension at 72°C for 40 s. The final elongation step was at 72°C for 10 min. Negative controls containing sterile water instead of template were included in each set of PCR. The PCR products were checked visually in an ethidium bromide-stained 2% agarose gel and then stored at –20°C. DGGE was performed on an INGENYphorU-2 electrophoresis system (INGENY, The Netherlands) as previously described [38] with a standard sample known to cover a wide range of bands loaded at least twice per gel.

The DGGE bands were defined manually and their numbers and positions were analyzed with BioNumerics 4.5 (Applied Maths NV, Belgium). Levels of similarity between DGGE profiles were calculated using the Dice similarity coefficient and the UPGMA clustering algorithm. Principal component analysis (PCA) was then performed for three-dimensional visualization of DGGE data and for extraction of PC1, PC2 and PC3 component values in order to carry out statistical comparison of parameters potentially affecting bacterial communities, such as copepod species, treatment, and time, as described previously [39, 40]. Analysis of variance, ANOVA (SigmaPlot 12.5, Systat Software, CA), was used to compare parameters based on PC1, PC2 and PC3 values from the PCA plot followed by pairwise multiple comparison procedures (Holm-Šídák method).

### 454-pyrosequencing and data treatment

Tag-encoded 454-pyrosequencing of 16S rRNA gene V3–V4 region amplicons (466 bp) from homogenized copepod/bacteria samples was done at the National High-throughput DNA Sequencing Centre, University of Copenhagen, Denmark, according to the manufacturer’s (Roche) instructions using the 341F (50-CCTAYGGGRBGCASCAG-30) and 806R (50-GGACTACNNGGGTATCTAAT-30) primers as described earlier [41]. The raw dataset was analyzed using the Quantitative Insight Into Microbial Ecology (QIIME 1.7) open software [42]; quality control of sequences, denoising and chimera removal were carried out as previously described [41]. Clustering of sequences was made with a 97% similarity cut-off using UCLUST [43] and OTU (operational taxonomic unit) picking was performed using the 16S rRNA gene database Greengenes (97% similarity, version 12.10) [44, 45].

### Statistical Analysis

For all statistical analyses samples were subsampled using a minimum cut-off of 1405 sequences (approx. 88% of the second most indigent sample), samples gathering a number of sequences below this threshold were not used in further analysis. Alpha Diversity measurements were computed using 10 rarified OTU tables and expressed as observed species (97% similarity OTUs), and their comparison was made with a non-parametric *t*-test (Monte Carlo, 999 permutations). PCoA plots were generated using the Jackknife Beta Diversity workflow (10 distance metrics were calculated based on 10 OTU tables). Analysis of similarities (ANOSIM) was used to determine differences within weighted and unweighted UniFrac distance matrices. Furthermore, differences in the relative distribution of taxa across categories were assessed with ANOVA (1,000 subsampled OTU tables).

## Results

### Development in chemical and physical properties of the system

The sampling campaign took place during the last month of summer, resulting in the water temperature in all tanks increasing from 18 to 20°C in mid-August followed by a decline to 16–17°C. The primary production and resulting turbidity was markedly higher in tanks with nutrients added (‘pulse’ and ‘full’ treatments) as evidenced by Secchi depths decreasing to 0.9 m, whereas Secchi depths in control tanks were never below 2.3 m. This increased primary production led to elevated O_2_ concentrations and pH in pulse and full treatments, whereas pH and O_2_ levels were more stable in the control treatment. O_2_ concentrations in pulse and full treatments reached 18.2 and 21.7 mg mL^-1^, respectively, in comparison to an O_2_ concentration of maximum 11.1 mg mL^-1^ in the control tanks. Likewise, pH in control tanks stayed almost constant at 8.3–8.5 but increased to pH 9.2 and 9.5 in the ‘pulse’ and ‘full’ treatments, respectively. Biotic and abiotic characteristics of plankton communities in the experimental tanks will be presented in more details elsewhere [32].

### Visualization of bacteria associated with copepods

#### SEM

**Fig 1:**
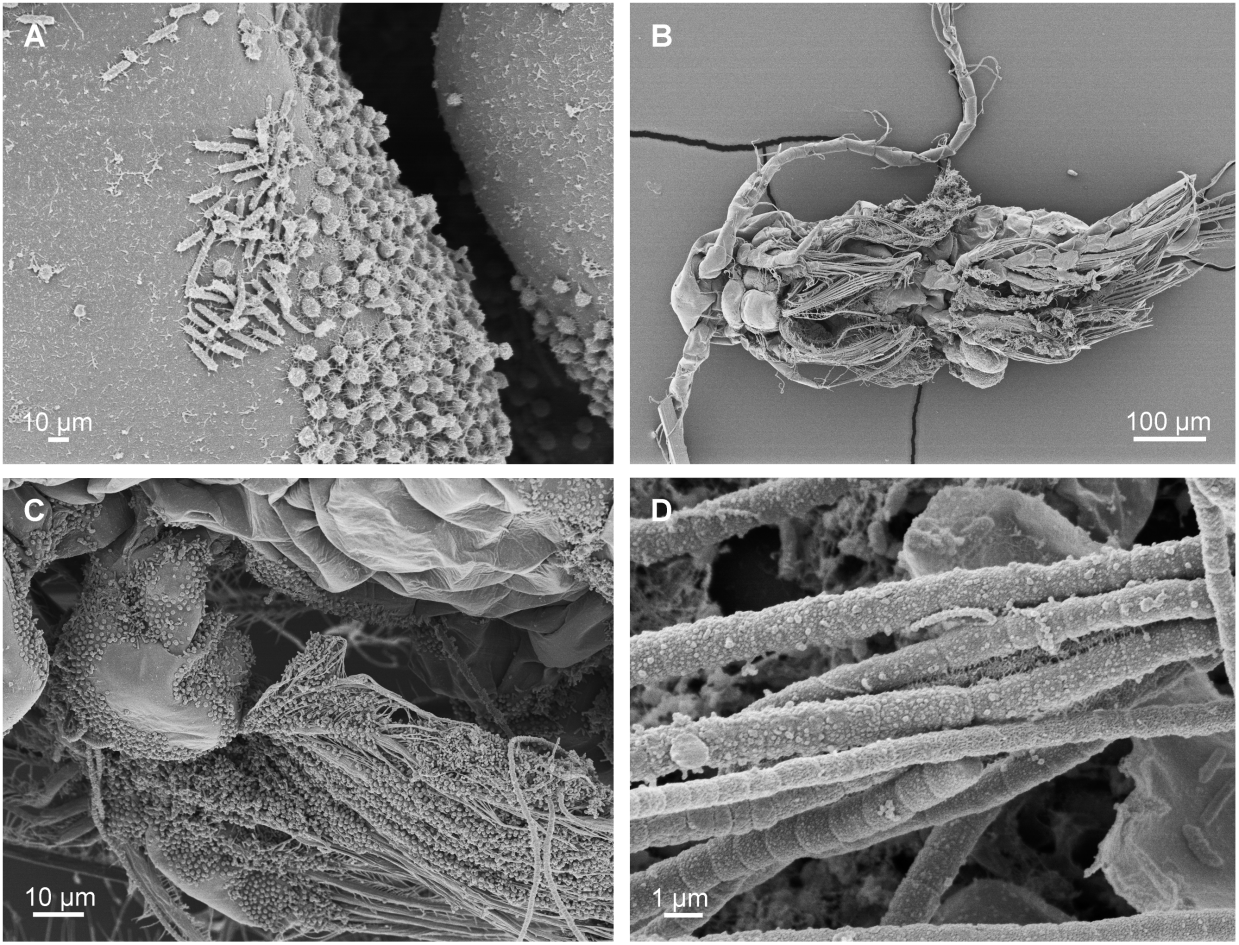
SEM micrographs of *Acartia tonsa* with attached bacteria. A. Specimen from outdoor tank depicting rod-shaped and coccoid bacteria morphotypes. B-D. Specimen from laboratory copepod culture. B. Overview of ventral side of copepod. C. Details of feeding appendages heavily colonized by bacteria. D. Details of (B) showing cluster of filamentous bacteria.

A dense cover of bacteria of different morphotypes was present on the copepods from the outdoor production ponds (Fig 1). The dominating morphotypes appeared to be coccoid with a diameter of approximately 0.75 μm and rod-shaped bacteria with a length of up to 1 μm (Fig 1A). Bacteria were primarily attached near junctions and furrows in the prosome and on basis and endites of limbs (Fig 1A). For comparison, *A*. *tonsa* individuals were collected from a long-term laboratory copepod culture (Fig 1B–1D). In the latter case, a pronounced presence of bacteria was seen the exoskeleton as well as on setae of swimming legs and feeding appendages. In addition, clusters of filamentous bacteria were adhered to the copepods (Fig 1D).

#### In-situ hybridization

**Fig 2:**
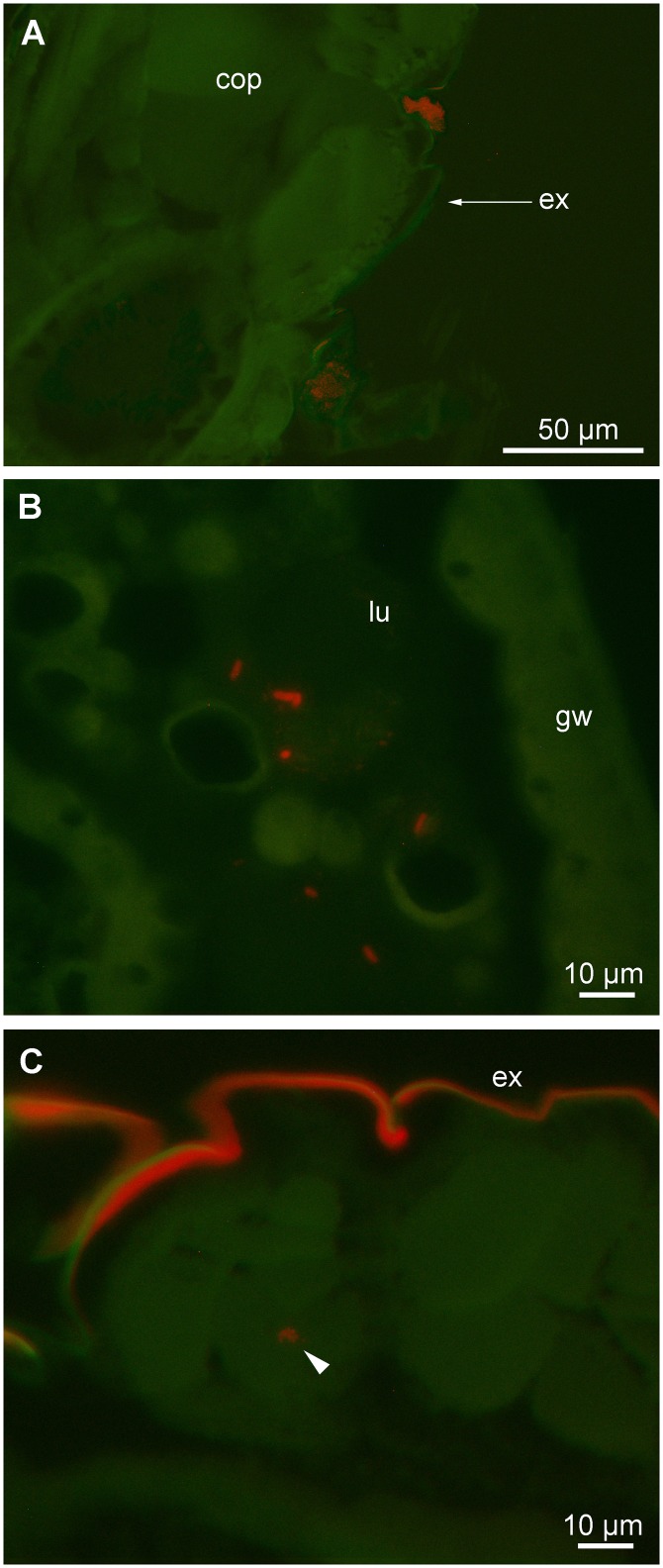
Fluorescent *in situ* hybridization of copepod (*C*. *hamatus*) thin sections stained with the universal bacterial Cy3-labelled probe EUB338. Pictures are overlays of two photographs made with filter sets for Cy3 (red) and autofluorescence (green), respectively. A. Exoskeleton with clusters of bacteria attached at outer part of depressions. B. Section of gut showing isolated rod-shaped bacteria. C. Potential bacteria (arrowhead) in skeletal muscle. Note that exoskeleton typically fluoresces with similar colour as the Cy3-labelled probe. Cop = copepod; ex = exoskeleton; lu = gut lumen; gw = gut wall.

Fluorescence microscopy confirmed findings by SEM that bacteria were attached in large clusters externally on the prosome, particularly around furrows and junctions in the exoskeleton (Fig 2A). Bacteria could also be detected in the lumen of the gut (Fig 2B) and in a couple of occasions the fluorescent signal of assumed bacterial cells was present in skeletal musculature (Fig 2C). Unlike the rare occurrence of internal bacteria, externally attached bacteria could be identified in all 25 specimens that were investigated by semi-thin sectioning and *in-situ* hybridization.

### Bacterial community composition as determined by DGGE

**Fig 3:**
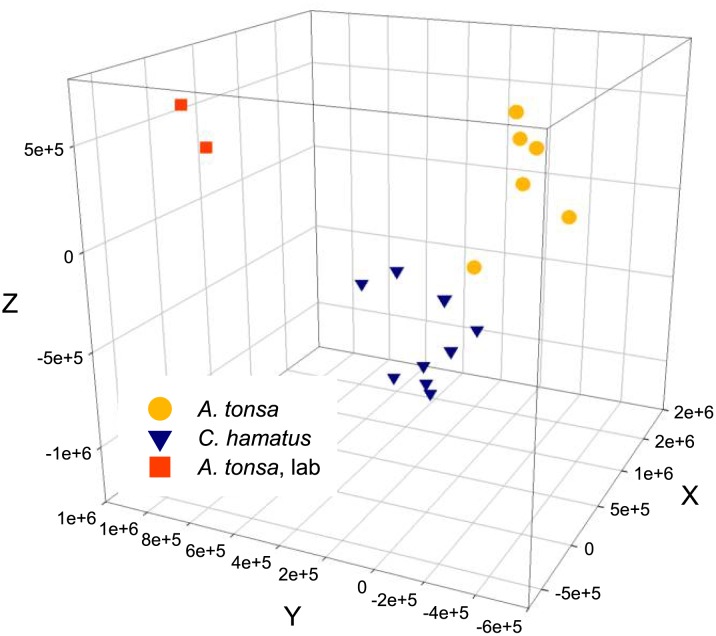
Principal component analysis based on DGGE profiles of the V3-region of the 16S rRNA gene (bacteria) associated two copepod species (*Acartia tonsa* and *Centropages hamatus*) in an extensive production system with 9 separate tanks and one laboratory culture.

Based on DGGE band patterns it was not possible to detect any trends or difference within the bacterial community structure as a function of the different tank treatments (i.e. control vs. ‘pulse’ or ‘full’ doses of nutrient addition). On the other hand, there was a distinct dissimilarity between the assemblages of bacterial genotypes present in the samples of the two copepod species (*A*. *tonsa* and *C*. *hamatus*) from the extensive copepod production tanks. Presenting the DGGE-derived data as a 3D principal component analysis (PCA) plot showed a clear overall separation of the samples from the two copepod species (Fig 3). In addition, the samples from laboratory-reared *A*. *tonsa* separated out in a third, distant cluster. Separation was statistically significant for all three types of samples for PC2 (*p* < 0.001) as well as for PC3 for both laboratory *A*. *tonsa* and outdoor in comparison with *C*. *hamatus* (*p* < 0.001, Fig 3).

### 454-pyrosequencing

Twenty-eight samples subjected to 454-pyrosequencing yielded 556,096 reads. After pre-processing of the dataset (quality control, sorting, trimming, denoising and filtering of chimeric sequences) 452,983 reads were obtained. The mean sequence length was 468 bp (SD 12 bp) and the average number of reads per sample was 16,178 (max = 130,594, min = 562). Samples with a number of sequences below 1,405 were not used in further analyses (n = 2). The metadata has been deposited in the European Nucleotide Archive (ENA) under accession number PRJEB8785 and additional metadata-information is available in S1 Table.

#### Dominant bacteria OTUs associated with the copepods

**Fig 4:**
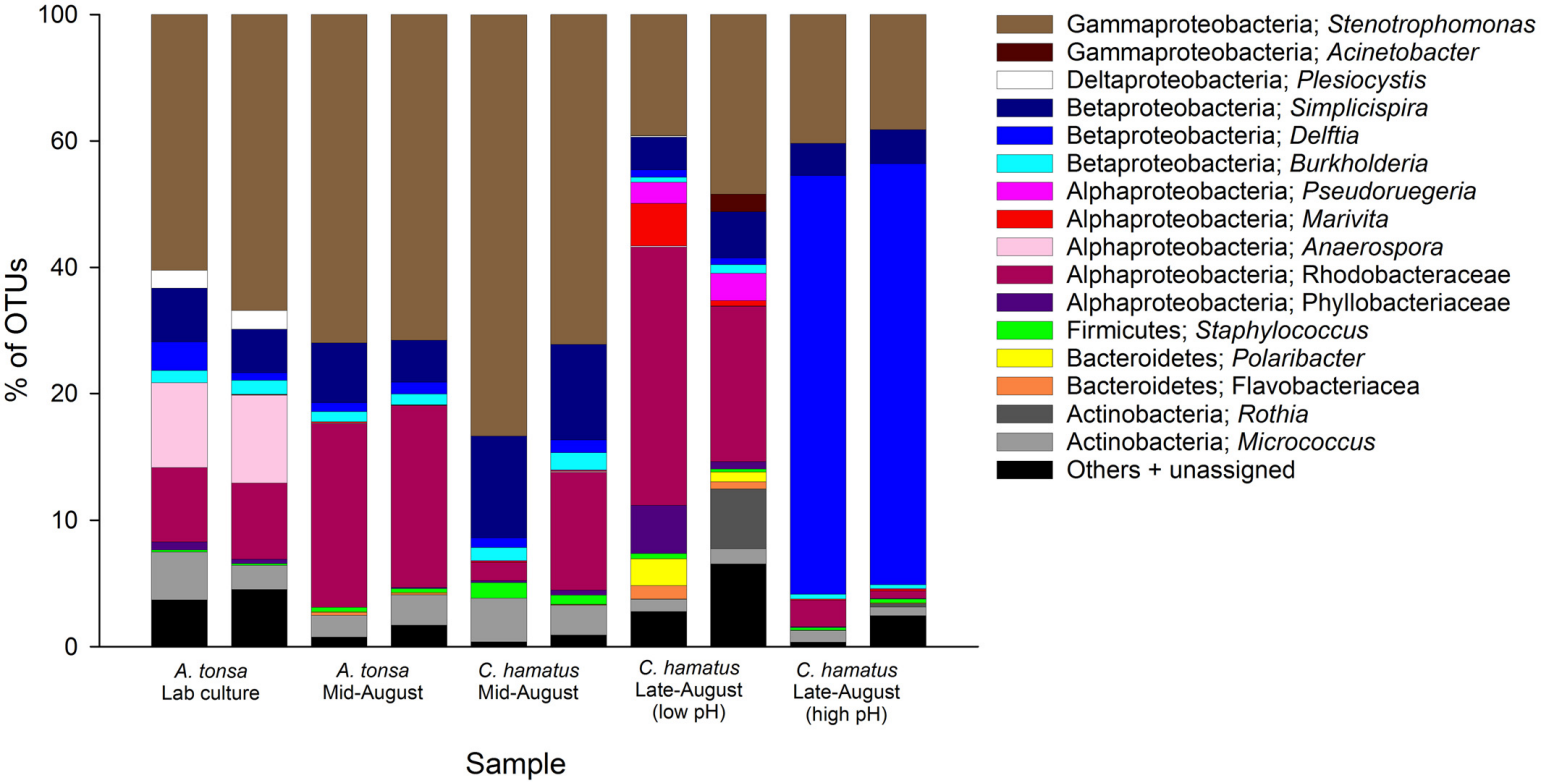
Relative distribution of bacterial 16s rRNA gene sequence reads in 10 out of a total of 28 454/FLX-based 16S rRNA gene amplicon sequencing samples. Samples are (in pairs): *Acartia tonsa* from laboratory culture (left two columns), and *A*. *tonsa* and *Centropages hamatus* from large-scale outdoor cultivation tanks. Outdoor samples are from two different dates in August 2012, representing different pH-levels in cultivation tanks. See text for detailed information. *) The left *A*. *tonsa* lab culture sample yielded less than 1,405 reads and both of these were, therefore, excluded from subsequent statistical analysis. OUTs representing less than 2% of total bacteria sequences are included in the group ‘Others + unassigned’.

The overall numerically dominating bacterial OTUs were from the phylum Proteobacteria of which the class Gammaproteobacteria were most frequently represented followed by Alphaproteobacteria and Betaproteobacteria. The phyla Actinobacteria, Bacteroidetes, and Firmicutes were present in all samples but together never comprised more than 10% of OTUs. Cyanobacteria and Verrucomicrobia were detectable in some samples, but never exceeded 0.2% of OTUs. On the OTU level, the overall most abundant taxon was *Stenotrophomonas* (Gammaproteobacteria; family Xanthomonadaceae), being present in all samples (Fig 4) and accounting for 13–71% of OTUs. OTUs assigned to Rhodobacteraceae (Alphaproteobacteria) and *Simplicispira* (Betaproteobacteria; Comamonadaceae) were also present in all samples, comprising 0.5–29% and 3.2–16% of all OTUs, respectively. The majority of the most abundant OTUs were present in all samples in variable distribution, with the exception of *Plesiocystis* (Deltaproteobacteria; family Nannocystaceae) which was present in laboratory *A*. *tonsa* samples (2.8–3% abundance) but virtually absent in outdoor samples. *Anaerospora* (Alphaproteobacteria; family Rhodobacteraceae) was evidently more abundant in laboratory *A*. *tonsa* samples (13–14%, Fig 4), but this genus was also present in most other samples albeit in low numbers (≤ 0.5%). Interestingly, one abundant Rhodobacteraceae OTU was highly similar (99%) to Rhodobacteraceae sequences found associated with copepods in two previous independent studies on microbiomes of marine copepods (GenBank accession numbers DQ839261 and JX435721) and one sequence from a bacteria associated with marine phytoplankton (AY684334).

#### Characterization of *C*. *hamatus* and *A*. *tonsa* microbiomes

**Fig 5:**
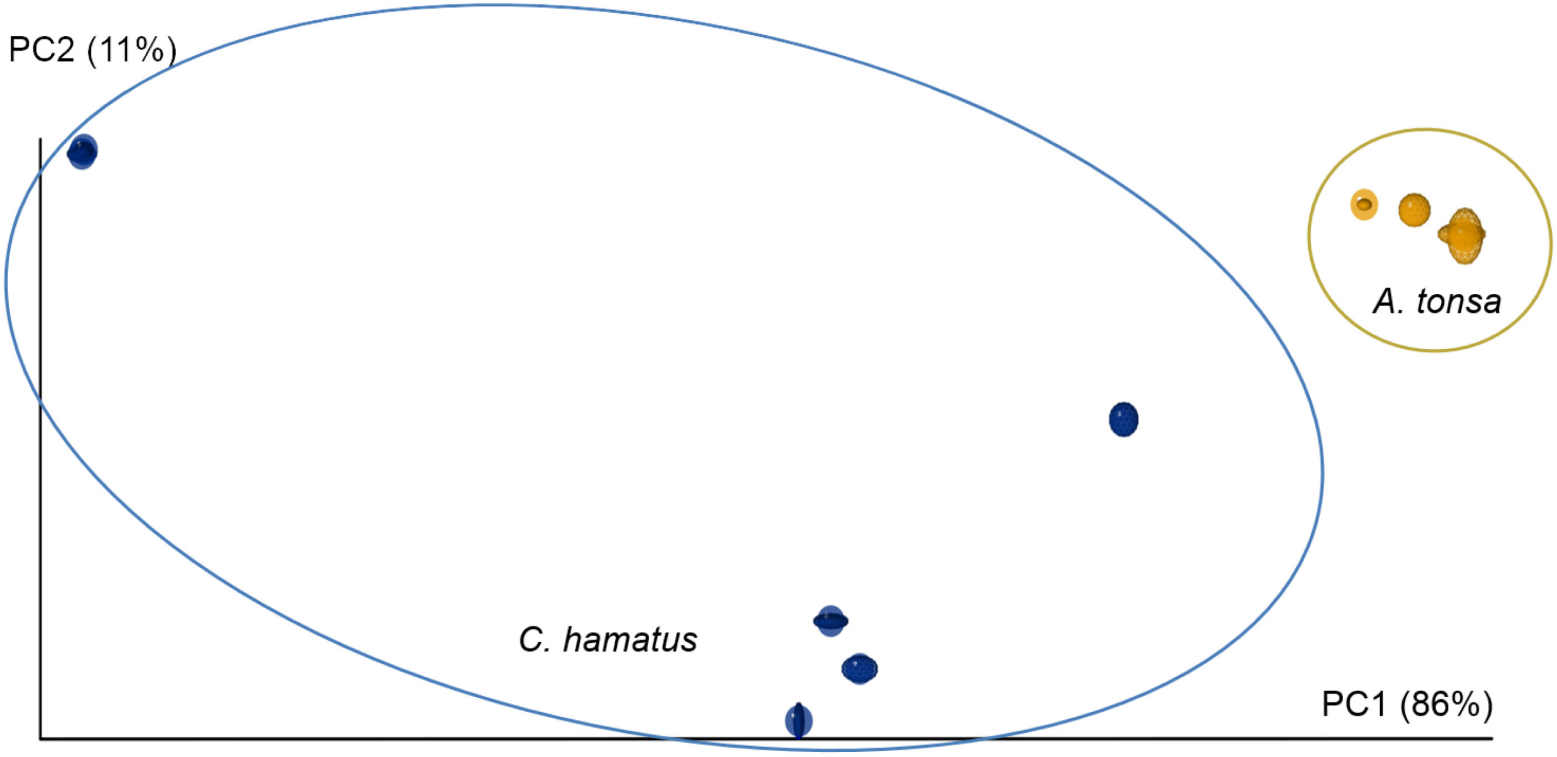
PCoA plot representing the microbiomes of *Centropages hamatus* (blue) and *Acartia tonsa* (orange). The plot is based on distance matrices determined by the Jackknife Beta Diversity workflow. Differences between microbiomes were assessed using ANOSIM (*p* = 0.047, *r* = 0.34). The ellipsoids depict the degree of variation for each sample.

**Fig 6:**
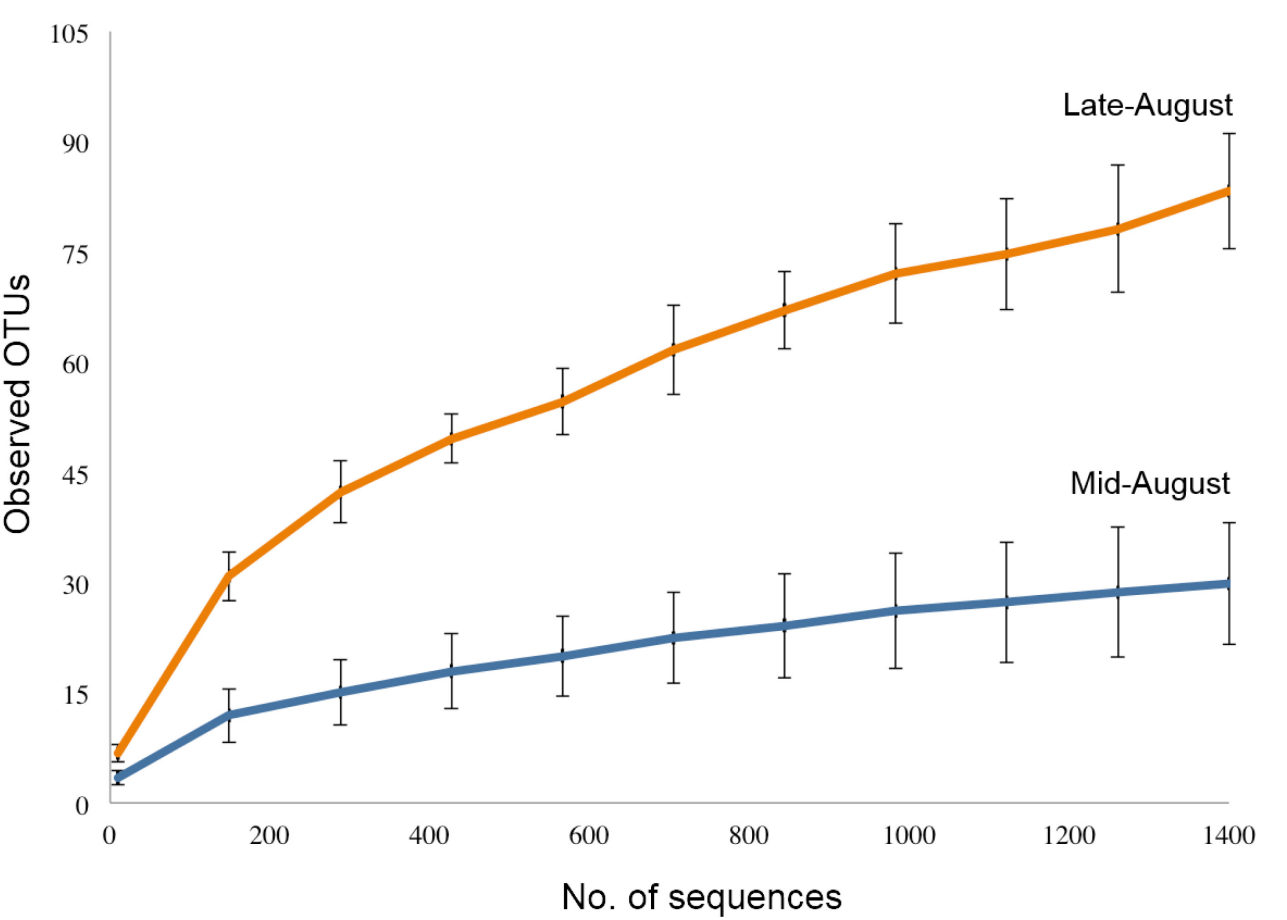
Rarefactions curves estimating the number of observed OTUs/species for the microbiomes of *Centropages hamatus* collected on Mid-August (blue line) and Late-August (orange line). Rarefactions were estimated using 1,405 sequences with 97% sequence identity threshold.

The microbiomes of *C*. *hamatus* and *A*. *tonsa* did not differ in the number of estimated observed species (*t*-test, *p* ≥ 0.05) and for each microbiome 30 (SD 8) and 34 (SD 3) species-level (97% similarity) OTUs were observed, respectively. Weighted PCoA UniFrac (Fig 5), on the other hand, showed clear separation of both copepods’ microbiomes (ANOSIM, *p* < 0.05, weighted *r* = 0.46). Both microbiomes displayed changes in the relative abundance of three OTUs (Table 1), where an unclassified genus of the Rhodobacteraceae family had the largest shift in relative distribution. Samples of laboratory-reared *A*. *tonsa* were not included in this analysis, because only a single of these samples yielded numbers of OTUs exceeding the cut-off level of 1,405 sequences. Within *C*. *hamatus*, differences in microbiome diversity were observed (*t*-test, *p* ≥ 0.05) over time (those collected on Mid-August, 13^th^-16^th^ and on Late-August, 25^th^-29^th^). The number of observed species estimated in crustaceans collected in Mid-August and Late-August was 30 (SD = 8) and 83 (SD = 7), respectively (Fig 6). These microbiomes were clearly separated by analysis of similarities in weighted and unweighted PCoA UniFrac (S1 Fig) along with a reduction in the relative abundance of six OTUs on copepods collected on Late-August (Table 2).

**Table 1:** Differences in operational taxonomic unit abundance between *C*. *hamatus* and *A*. *tonsa* microbiomes determined by ANOVA.

Bacteria					Relative distribution		Significance*	
Phylum	Class	Order	Family	Genus	*C*. *hamatus*	*A*. *tonsa*	*p*-value	*q*-value
Proteobacteria	Alphaproteobacteria	Rhodobacterales	Rhodobacteraceae	Unclassified	28.5%	5.8%	<0.001	0.003
Bacteroidetes	Flavobacteriia	Flavobacteriales	Unclassified	Unclassified	0.5%	<0.1%	0.001	0.009
Firmicutes	Bacilli	Bacillales	Staphylococcaceae	*Staphylococcus*	0.8%	1.7%	0.046	0.641

* p- and *q-*values were determined with ANOVA; *q-*values represent Bonferroni correction; ANOVA was performed using 1,000 subsampled OTU tables.

**Table 2:** Shifts in the relative distribution of operational taxonomic units between the microbiomes of *C*. *hamatus* collected on Mid-August and Late-August.

Bacteria					Relative distribution		Significance*	
Phylum	Class	Order	Family	Genus	Mid-August	Late-August	*p*-value	*q*-value
Bacteroidetes	Flavobacteriia	Flavobacteriales	Flavobacteriaceae	Unclassified	1.6%	<0.1%	0.001	0.017
Proteobacteria	Alphaproteobacteria	Rhodobacterales	Rhodobacteraceae	Unclassified	36.7%	5.8%	0.003	0.035
Bacteroidetes	Flavobacteriia	Flavobacteriales	Flavobacteriaceae	*Polaribacter*	2.9%	0.1%	0.005	0.071
Other	Other	Other	Other	Other	0.6%	0.1%	0.029	0.376
Proteobacteria	Alphaproteobacteria	Rhodobacterales	Rhodobacteraceae	*Marivita*	3.6%	0.2%	0.041	0.528
Proteobacteria	Alphaproteobacteria	Rhizobiales	Phyllobacteriaceae	Unclassified	4.4%	0.4%	0.045	0.587

* p- and *q-*values were determined with ANOVA; *q-* values represent Bonferroni correction; ANOVA was performed using 1,000 subsampled OTU tables.

#### Microbiome of *C*. *hamatus* under low/high pH conditions

**Fig 7:**
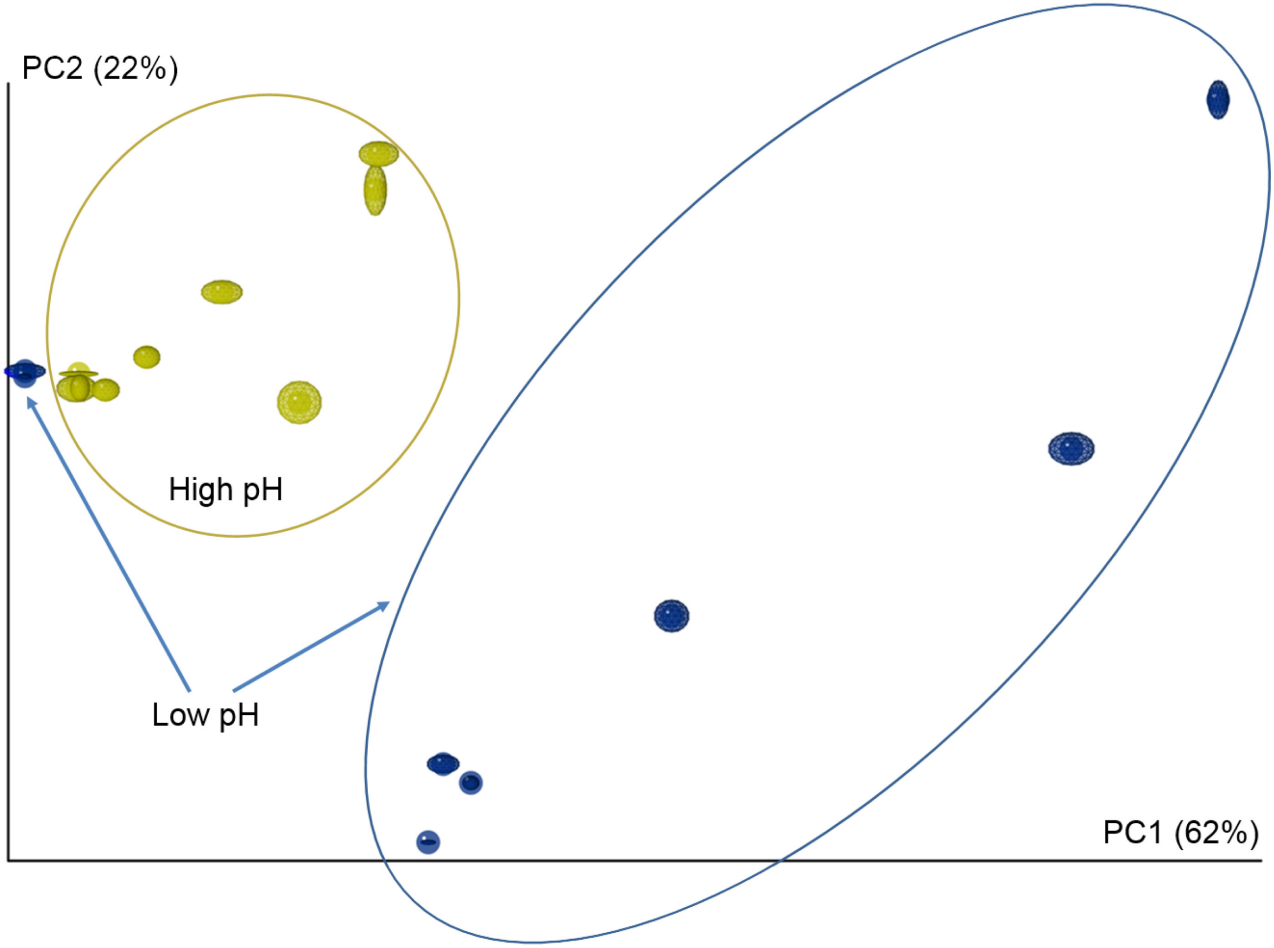
PCoA plot of the microbiomes of *Centropages hamatus* under low (blue) and high pH (yellow). The plot is based on distance matrices determined by the Jackknife Beta Diversity workflow. Differences between microbiomes were assessed using ANOSIM (*p* = 0.005, *r* = 0.34). The ellipsoids depict the degree of variation for each sample.

The pH of the water in the tanks of the ‘pulse’ and ‘full’ treatments increased from 8.3 to 9.8 during the study period. Relating the copepod microbiomes to the three different nutrient treatment regimes (i.e. control, ‘pulse’, and ‘full’) showed a tendency of the ‘pulse’ and ‘full’ treatments to cluster together and separated from the control treatments (data not shown). Different nutrient regimes will alter biological and chemical parameters in the culture tanks and the copepod microbiomes were, therefore, compared in relation to parameters measured in the tanks, such as pH, O_2_-concentration, Secchi depth, temperature, and salinity. There was no significant effect to be found of temperature, salinity, and Secchi depth on the copepod microbiomes. On the other hand, after categorization of pH values, clustering of microbiomes due to high pH (≥ 8.8) and low pH (< 8.8) was observed in weighted PCoA UniFrac (Fig 7; ANOSIM, *p* < 0.01, *r* = 0.34). The relative abundance of six genera was significantly different between these pH groups (Table 3). The genus *Delftia* constituted more than 60% of the *C*. *hamatus* microbiome (relative distribution) in high pH waters, whereas *Simplicispira*, *Stenotrophomonas*, *Burkholderia*, *Staphylococcus* and *Micrococcus* together accounted for 60% or more of the copepods microbiome in low pH waters. A PCoA based on O_2_-concentrations yielded a similar pattern (data not shown).

**Table 3:** Variations in operational taxonomic unit composition between the microbiomes of *C*. *hamatus* collected under low pH (<8.8) and high pH (≥8.8) conditions.

Bacteria					Relative distribution		Significance*	
Phylum	Class	Order	Family	Genus	Low pH	High pH	*p*-value	*q*-value
Proteobacteria	Betaproteobacteria	Burkholderiales	Comamonadaceae	*Delftia*	18.9%	61.3%	0.002	0.025
Proteobacteria	Betaproteobacteria	Burkholderiales	Comamonadaceae	*Simplicispira*	10.8%	4.7%	0.005	0.070
Proteobacteria	Gammaproteobacteria	Xanthomonadales	Xanthomonadaceae	*Stenotrophomonas*	41.3%	18.3%	0.007	0.103
Proteobacteria	Betaproteobacteria	Burkholderiales	Burkholderiaceae	*Burkholderia*	1.8%	0.7%	0.009	0.130
Firmicutes	Bacilli	Bacillales	Staphylococcaceae	*Staphylococcus*	1.4%	0.6%	0.017	0.240
Actinobacteria	Actinobacteria	Actinomycetales	Micrococcaceae	*Micrococcus*	3.6%	1.5%	0.019	0.262

* p- and *q-*values were determined with ANOVA; *q-* values represent Bonferroni correction; ANOVA was performed using 1,000 subsampled OTU tables.

## Discussion

### Visualization of bacteria

Bacteria associated with live copepods are generally considered to be attached as epibionts to the copepod exoskeleton [46, 47], but it has also been documented through microelectrode oxygen measurements that bacteria thrive in the gut of copepods [4]. Based on *in-situ* hybridization it is confirmed here that bacteria are plentiful on the copepod exoskeleton and that bacteria are also present in the gut (Fig 2). Bacteria were detected on the exoskeleton on most investigated copepods (n = 25), but gut bacteria were found in only three specimens. The occurrence of bacteria in the lumen of the gut may, however, have been underestimated, because copepods embedded in paraffin tend to lose content of the gut lumen [48]. The presence of numerous bacteria of varying morphology attached the exoskeleton was furthermore demonstrated through SEM (Fig 1), confirming previous findings by other authors [19]. The potential ecological significance of bacteria attached to copepods is largely unknown, but these bacteria do cause exoskeletal scars [49] and a heavy colonization by bacteria may weaken the host’s exoskeleton. Solid proof of bacteria infecting copepod tissue was not obtained in the present study except from a single case of what appeared to be bacteria in a skeletal muscle (Fig 2C). Nevertheless, internal bacteria assumed to be infectious and potentially pathogenic do occur in the tissue of marine copepods [50], but the magnitude of this copepod-bacteria association has still to be explored.

Laboratory cultured *A*. *tonsa* were densely colonized with bacteria on the exoskeleton as well as on and around feeding appendages (Fig 1B). It appeared that laboratory cultured *A*. *tonsa* were more densely covered with bacteria than the copepods from the outdoor extensive system. While this is only a qualitative observation, it seems plausible that bacteria will more effectively colonize copepods in intensive, closed systems, because such systems are likely to have higher contents of organic matter and support fewer protozoan grazers able to exploit and check the bacteria population. In fact, mass culturing of copepods appears to offer optimal conditions not only for epibiotic bacteria but also for epibiotic protozoans [51]. Clearly, bacterial colonization will depend on culture conditions, feeding scheme, age of cultures etc., which in turn will affect bacterial growth conditions and the composition of protozoan community feeding upon bacteria.

### Characterization of microbiomes

The present study is among the first to characterize microbiomes of marine copepods by use of 2^nd^ generation sequencing methodology. Hence, comparison of bacterial species composition and richness can be done primarily with corresponding studies based on DGGE, Sanger sequencing or culture-dependent techniques. In general, DGGE based surveys have shown Alphaproteobacteria and Gammaproteobacteria to be the predominating groups of bacteria in live marine copepods [3, 21–23] with minor contributions by Betaproteobacteria, Actinobacteria, Verrucomicrobia, Firmicutes, and Bacteroidetes [3, 22]. Culture-dependent studies have, however, pointed toward Gammaproteobacteria being numerically dominant [12, 20].

The present analysis confirms the predominance of Alphaproteobacteria and Gammaproteobacteria as well as the presence of Actinobacteria, Verrucomicrobia, Firmicutes, and Bacteroidetes in relatively low abundance (Fig 2). It is noteworthy, however, that Betaproteobacteria (represented primarily by the genus *Stenotrophomonas*) accounted for up to 71% of all OTUs associated with both outdoor tanks *A*. *tonsa* and *C*. *hamatus* and laboratory-reared *A*. *tonsa*. This genus has, just as some of the other predominant OTUs, *Simplicispira* and *Anaerospora*, not previously been observed in association with marine zooplankton. Another OTU assigned to *Delftia* in the present study was also detected in the microbiome of North Sea copepods through DGGE and Sanger sequencing-based methodology [22]. It is important to keep in mind, however, that different primer sets were used in the aforementioned DGGE-based study and the present 454-pyrosequencing-based study and this could potentially account for some of the observed differences in the microbiomes.

In the present study, a minimum of 2 and a maximum of 24 DGGE bands were discernible for each sample (a total of 48 samples). This permitted a clear distinction between the microbiomes of *A*. *tonsa* and *C*. *hamatus* from outdoor tanks and laboratory-reared *A*. *tonsa*, respectively (Fig 3). In the DGGE-based PCA (Fig 3), a clear separation of band patterns was found between *A*. *tonsa* and *C*. *hamatus* sampled at the same time and in the same cultivation tanks. It is less surprising that laboratory-reared *A*. *tonsa* clustered distantly from the two species sampled in outdoor tanks, since environments in laboratory cultures and outdoor tanks were likely to be distinct. Comparable studies of differences in copepod microbiomes across copepod species have not uncovered any general trend as to whether different copepod species host species-specific microbiomes. A tendency of differences in species composition in the microbiomes of *Temora* sp. and *Acartia* sp. in the North Sea has been observed [26]. However, in a following comprehensive study, no difference in the composition of the microbiomes of four North Sea copepod species was found and no seasonal differences in these microbiomes were evident [22]. On the opposite, a study from the Godthåbsfjord in Greenland [3] showed apparent differences in the microbiome of the Artic copepod species *Calanus finmarchicus* and *Metridia longa*. Unfortunately, these results were challenged by the presence of large numbers of phytoplankton chloroplast rDNA sequences in the samples that probably disguised bacterial sequences and the issue of host-specificic microbiomes was, therefore, not satisfactorily resolved.

The application of 454-pyrosequencing yielded up to 83 different bacterial species level OTUs for a single type of copepod sample (*C*. *hamatus* Late-August, Fig 6), despite the cut-off threshold being set relatively low at 1,405 sequences per sample (corresponding to approx. 88% of the second most indigent sample). This allowed for observing a shift in the microbiome of a single copepod species over a time period of 2 weeks (Fig 7, Table 3). Higher bacterial diversity over time may be a function of higher eutrophication of tanks with resulting higher production of organic matter. Unlike the DGGE-analysis in which *A*. *tonsa* and *C*. *hamatus* displayed different band patterns, i.e. some bacteria were present in one species, but not in the other (and visa versa), the 454-pyrosequencing analysis showed that the identities of bacteria OTUs did not differ significantly between copepod species, but there were statistically significant differences in the proportions of bacteria species (Fig 5, Table 1). There may seem to be a discrepancy between these two analyses. However, DGGE yields no quantitative information except from the possibility to subjectively differentiate between band intensities. The 454-pyrosequencing, on the other hand, is more sensitive than DGGE and picks up many more OTUs, including the less abundant ones, some of which will only produce faint or invisible bands in the DGGE gel.

The copepod biome is not separated from the bacterial community of the ambient water, but is characterized by an active exchange of bacteria between water and copepods [21], and the same bacteria are often present in the water and in association with copepods, albeit in different proportions [21, 27]. Yet, copepods may constitute microenvironments that favour certain bacteria, and in some cases copepod microbiomes do differ both qualitatively and proportionally from the bacteria community in ambient seawater [3, 27]. One mechanism by which host-specific bacteria may be distributed to different hosts is through feeding [23] and, in particular, through preferential feeding on certain phytoplankton species, since these may themselves be associated with different bacteria [52]. A general picture is thus forming that the copepod microbiome is not merely a reflection of the bacterial community in the ambient water, but rather copepods constitute microenvironments that support specific niches for certain bacteria species. This is supported by the fact that a Rhodobacteraceae OTU observed in the present study has previously been found in association with copepods [21, 22], suggesting that this OTU represents a bacterial species adapted to growth in close association with marine plankton.

Changes in bacterial community could be attributed mainly to chemical factors, i.e. O_2_ and/or pH. The increase in pH and O_2_-concentration in outdoor tanks resulted in a significant shift in copepod microbiomes (Figs 4 and 7, Table 3) that was primarily driven by the increased occurrence of the Betaproteobacteria *Delftia* sp. in samples from tanks with water exceeding pH 8.8. Other bacteria, such as *Simplicispira* and *Stenotrophomonas*, became less abundant. The increasing pH (up to 9.5) was a natural consequence of high primary production leading to increased O_2_-concentration and lowered CO_2_-concentration with resulting higher pH. Based on the available data it is thus not possible to determine whether it was pH or O_2_ that was the driving force for this shift in the microbiome. However, minor changes in CO_2_-levels and/or pH can affect the physiology of phytoplankton and zooplankton organisms [53, 54] and can cause shifts in bacteria species composition [55]. It is, therefore, feasible that the lead factor responsible for the shift in the copepod microbiome was pH.

In conclusion, DGGE and 454-pyrosequencing revealed differences in copepod microbiomes in terms of host-specificity (*A*. *tonsa* vs. *C*. *hamatus*) and, in particular, as function of time within a single species (*C*. *hamatus*). Host specificity was most evident in the DGGE analysis in which evident results may, however, be a consequence of some bacteria species not being satisfactorily visualized by use of this technique. Nonetheless, 454-pyrosequencing did reveal quantitative differences in the species-rich microbiomes of the two copepod species living side-by-side in the same environment. Both DGGE and 454-pyrosequencing showed clear differences in the microbiomes of *A*. *tonsa* from outdoor tanks and laboratory cultures; in both cases several OTUs were present in only one type of sample. Interestingly, however, certain bacterial OTUs (such as *Stenotrophomonas*) were consistently present in all copepod microbiomes, independent of copepod species and cultivation type. The most important factors found to affect the composition of copepod microbiomes were abiotic parameters affecting bacteria growth conditions (i.e. pH and/or O_2_-concentration). This demonstrates a strong overall dependence of the copepod microbiome on ambient water conditions and the microenvironment for bacteria presented by copepods is thus not self-sustained, but is highly dependent on conditions prevailing in the water.

None of the most abundant OTUs of the present study matched the sequences of any known pathogenic bacteria of fish mariculture [56]. However, a single OTU had 99% similarity to *Pseudomonas anguilliseptica* (GenBank accession number AY870672) that causes hemorrhagic septicaemia in a number of fish species, including turbot [57]. This OTU was present in six outdoor tank samples with a maximum relative abundance of 0.2%. The existence of this OTU does, however, highlight the possibility of copepods being able to serve as reservoirs for potential fish-pathogenic bacteria in parallel to the case of copepods functioning as reservoir for the human-pathogenic *V*. *cholerae* [15]. The large and changing diversity of bacteria associated with copepods opens up for exploration of the role of copepod microbiomes; i.e. what is the role of associated bacteria and what are likely effects of changes in the microbiome. Such effects on copepods and potential pathogenicity of associated bacteria are yet to be investigated. In general, bacteria associated with copepods are considered passive symbionts, but infectious bacteria do occur [50] and epibiotic bacteria may exert physiological harm when colonizing the exoskeleton [49]. The identity of these potentially pathogenic bacteria is unknown since only basal morphology data is presented in that study, and it is thus not possible to link sequence-based identification of bacteria with the above observations. Even non-pathogenic bacteria in the microbiome may have important roles for their hosts through e.g. affecting such factors as growth, fecundity, and egg hatching rates and egg/nauplii survival. It will, therefore, be relevant in the future to examine copepods microbiomes in cultures under varying growth states as well as in wild copepods in populations of different growth conditions.

## Supporting Information

S1 Table Metadata of sequenced samples used for screening the influence of pH, host and season (Mid- and Late-August) on the copepod microbiomes. (DOCX)

S1 Fig The ß-diversity metrics clusters *Centropages hamatus’* microbiomes collected on Mid- and Late-August. A) Unweighted (ANOSIM p = 0.038, r = 0.89). B) Weighted (ANOSIM p = 0.039, r = 0.83). Light green depicts Mid-August and blue depicts Late-August. The degree of variation for each sample is represented by the ellipsoids.
(EPS)
