# Supplementary material for: Host-Specific and pH-Dependent Microbiomes of Copepods in an Extensive Rearing System
Source: PLoS One. 2015 Jul 13;10(7):e0132516. doi: 10.1371/journal.pone.0132516 (PMC4500450; doi:10.1371/journal.pone.0132516)
Supplement: S1 Table — (DOCX) [file pone.0132516.s001.docx]

S1 Table. Metadata of sequenced samples used for screening the influence of pH, host and season (Mid- and Late-August) on the copepod microbiomes.

| #SampleID | Species | pH_range | pH_mean | O2_range | O2_mean | Season |
| --- | --- | --- | --- | --- | --- | --- |
| AF..01 | Cent | below_8.8 | 8.3 | below_13 | 10.8 | Mid_August |
| AF..02 | Cent | below_8.8 | 8.3 | below_13 | 9.7 | Mid_August |
| AF..03 | Cent | above_8.8 | 8.8 | above_13 | 15.1 | Mid_August |
| AF..04 | Cent | above_8.8 | 8.8 | above_13 | 15.1 | Mid_August |
| AF..05 | Cent | above_8.8 | 8.9 | above_13 | 18.3 | Mid_August |
| AF..06 | Cent | above_8.8 | 8.8 | above_13 | 17.3 | Mid_August |
| AF..07 | Cent | above_8.8 | 8.8 | above_13 | 17 | Mid_August |
| AF..08 | Cent | below_8.8 | 8.4 | below_13 | 10.5 | Mid_August |
| AF..09 | Cent | below_8.8 | 8.4 | below_13 | 10.5 | Mid_August |
| AF..10 | Cent | below_8.8 | 8.4 | below_13 | 10.5 | Mid_August |
| AF..11 | Cent | below_8.8 | 8.4 | below_13 | 10.5 | Mid_August |
| AF..12 | Cent | above_8.8 | 9.4 | above_13 | 18.1 | Late_August |
| AF..13 | Cent | above_8.8 | 9.2 | above_13 | 16.1 | Late_August |
| AF..14 | Cent | above_8.8 | 9.1 | above_13 | 15.6 | Late_August |
| AF..15 | Cent | above_8.8 | 9.0 | above_13 | 15.4 | Late_August |
| AF..16 | Cent | below_8.8 | 8.5 | below_13 | 11 | Late_August |
| AF..17 | Cent | below_8.8 | 8.4 | below_13 | 9.8 | Late_August |
| AF..22 | Acar | below_8.8 | 8.3 | below_13 | 10.8 | Mid_August |
| AF..23 | Acar | below_8.8 | 8.3 | below_13 | 9.7 | Mid_August |
| AF..24 | Acar | above_8.8 | 8.8 | above_13 | 15.1 | Mid_August |
| AF..26 | Acar | below_8.8 | 8.4 | below_13 | 10.5 | Mid_August |
